# Innovative Partnership Between Primary Care, AAA, and Academia Connects 4Ms and Social Determinants of Health

**DOI:** 10.1093/geroni/igaa057.352

**Published:** 2020-12-16

**Authors:** Suzanne Leahy, Katie Ehlman, Lisa Maish, Brad Conrad, Jillian Hall, Ron Wells, Laura Holscher

**Affiliations:** 1 University of Southern Indiana, Evansville, United States; 2 Deaconess Clinic, Evansville, United States; 3 SWIRCA & More, Evanville, Indiana, United States; 4 Generations Area Agency on Aging, Vincennes, Indiana, United States

## Abstract

Nationally, there is a growing focus on addressing geriatric care in primary care settings. HRSA’s Geriatric Workforce Enhancement Program (GWEP) has called for academic and health system partners to develop a reciprocal, innovative, cross-sector partnership that includes primary care sites and community-based agencies serving older adults. Through the University of Southern Indiana’s GWEP, the College of Nursing and Health Professions, the Deaconess Health System, three primary care clinics, and two Area Agencies on Aging (AAA) have joined to transform the healthcare of older adults regionally, including rural residents in the 12-county area. Core to the project is a value-based care model that “embeds” AAA care managers in primary care clinics. Preliminary evaluation indicates early success in improving the healthcare of older adults at one primary clinic, where clinical teams have referred 64 older adult patients to the AAA care manager. Among these 64 patients, 80% were connected to supplemental, community-based health services; 22% to programs addressing housing and transportation; and, nearly 10% to a range of other services (e.g., job training; language and literacy; and technology). In addition to presenting limited data on referred patients and referral outcomes, the presentation will share copies of the AAA referral log, to illustrate how resources were categorized by SDOH and added to support integration of the 4Ms.

